# Contrasting population genetic patterns within the white-throated sparrow genome (*Zonotrichia albicollis*)

**DOI:** 10.1186/1471-2156-11-96

**Published:** 2010-10-28

**Authors:** Lynn Y Huynh, Donna L Maney, James W Thomas

**Affiliations:** 1 Graduate Program in Population Biology, Ecology and Evolution, Emory University, Atlanta, GA, USA; 2 Department of Human Genetics, Emory University School of Medicine, Atlanta, GA, USA; 3 Department of Psychology, Emory University, Atlanta, GA, USA

## Abstract

**Background:**

The level of nucleotide diversity observed across the genome is positively correlated with the local rate of recombination. Avian karyotypes are typified by large variation in chromosome size and the rate of recombination in birds has been shown to be negatively correlated with chromosome size. It has thus been predicted that nucleotide diversity is negatively correlated with chromosome size in aves. However, there is limited empirical evidence to support this prediction.

**Results:**

Here we sequenced 27 autosomal and 12 sex chromosome-linked loci in the white-throated sparrow (*Zonotrichia albicollis*) to quantify and compare patterns of recombination, linkage disequilibrium (LD), and genetic diversity across the genome of this North American songbird. Genetic diversity on the autosomes varied up to 8-fold, with the lowest diversity observed on the macrochromosomes and the highest diversity on the microchromosomes. Genetic diversity on the sex chromosomes was reduced compared to the autosomes, the most extreme difference being a ~300-fold difference between the W chromosome and the microchromosomes. LD and population structure associated with a common inversion polymorphism (ZAL2/2^m^) in this species were found to be atypical compared to other macrochromosomes, and nucleotide diversity within this inversion on the two chromosome arrangements was more similar to that observed on the Z chromosome.

**Conclusions:**

A negative correlation between nucleotide diversity and autosome size was observed in the white-throated sparrow genome, as well as low levels of diversity on the sex chromosomes comparable to those reported in other birds. The population structure and extended LD associated with the ZAL2/2^m ^chromosomal polymorphism are exceptional compared to the rest of the white-throated sparrow genome.

## Background

The genomic landscape is influenced by the combined interactions of mutation, recombination, natural selection, genetic drift, and demographics. A primary goal of the field of population genetics is to understand the role and relative importance of these forces in generating and maintaining genetic variation. Within a genome, signatures of these forces can be inferred by examining local levels and patterns of genetic variation. In general, purifying selection is the dominant selective force in molecular evolution and results in a reduction of genetic diversity as new variants are selectively removed from the population [1]. Reductions in diversity can also arise from positive directional selection, in which a particular variant is favored by natural selection and rises to fixation resulting in the loss of diversity, referred to as a selective sweep [2]. By contrast, other forms of positive selection, called balancing selection, can increase the amount of genetic diversity through heterozygote advantage or frequency-dependent selection [3]. Finally, genetic drift can randomly influence the evolutionary fate of new mutations regardless of their selective benefit, such that in smaller populations, variants are more likely to be lost due to drift [4].

While selection and drift may act upon a single variant, the rate of recombination will determine whether closely linked sites will also be affected. In genomic locations where recombination is absent or infrequent, there will be linkage disequilibrium (LD), or non-random association of genetic variants. If purifying selection removes a deleterious mutation, any variation in strong LD with that mutation will also be removed [5]. If positive selection fixes a beneficial mutation, any linked variation will also be fixed [2]. Together, background selection and genetic hitchhiking reduce overall levels of variation and inhibit the efficacy of selection because selection cannot act on mutations independently as a result of LD. This phenomenon is known as Hill-Robertson Interference or HRI [6]. When recombination is frequent, the impact of HRI is minimized and standing variation is less susceptible to loss due to selection at another site. Levels of nucleotide diversity are therefore positively correlated with local rates of recombination [7] which are known to be variable across a genome [8].

Most avian genomes are organized into several large macrochromosomes, several intermediate sized chromosomes and many tiny microchromosomes [9]. In chicken and zebra finch, the rate of recombination on microchromosomes is 5-10 fold greater than on macrochromosomes [9,10]. The higher rate of recombination on the microchromosomes is at least in part due to the one obligatory cross-over required per chromosome arm for proper chromosome segregation during meiosis. As such, microchromosomes experience a higher rate of recombination per base than larger macrochromosomes, thus recombination rate increases as chromosome size decreases [11]. In zebra finch, Backström *et al*. [10] also found a pronounced telomere effect such that recombination rates are highly elevated within < 20 Mb of the telomeres. Because microchromosome are generally < 20 Mb, their recombination landscape is uniformly elevated relative to the central regions of macro- and intermediate chromosomes [10]. Considering that smaller chromosomes have higher recombination rates, and that higher recombination rates are associated with increased nucleotide diversity, it is reasonable to expect that smaller chromosomes will harbor increased genetic variation [12], though this prediction has not been investigated in depth in avian genomes [13,14]. Furthermore, because recombination rate increases so dramatically toward the chromosome ends [10], intrachromosomal variation in nucleotide polymorphism may also be considerable with the highest diversity expected near the telomeres.

Polymorphism levels also differ between autosomes and sex chromosomes [15]. This phenomenon occurs because standing variation is directly proportional to the effective population size (N_e_), which varies between sex chromosomes and autosomes. In a population consisting of equal numbers of males (ZZ) and females (ZW), there are 4 copies of each autosome for every 3 Z chromosomes, and every 1 W chromosome. The expected ratio of N_e _under a neutral model between autosomes and the Z and W chromosomes is therefore 4:3:1 [15], and levels of nucleotide diversity are predicted to show the same relationship. Studies of avian sex chromosomes [16,17] have shown that the diversity on the Z and W is lower than expected based on relative N_e _when compared to autosomes, which is a pattern that has been widely observed in XY systems as well [15]. Sex chromosomes differ in their pattern of recombination: recombination is reduced on the Z compared to autosomes and absent in the non-recombining regions of the W. As a consequence of the patterns of recombination on the sex chromosomes, HRI will further reduce the genetic diversity of the Z and W, with the non-recombining region of the W chromosome experiencing the most dramatic reduction in genetic diversity [18].

The W chromosome is in complete linkage disequilibrium with the mitochondrial genome, which is also non-recombining. As a result, any selective events on the W will affect the mitochondrial genome and vice versa [19]. The linkage disequilibrium between the W and mitochrondrial genome combined with HRI likely contribute to diversity levels on the W chromosome being 100-fold lower than those observed on autosomes [16,17]. The Z chromosome may also experience a further reduction in genetic diversity due to the fact that two-thirds of the time it is passed through the male germ line. When there is high variation in male mating success, the N_e _of the Z chromosome will be reduced, as well as the amount of neutral standing variation [20,21]. Thus, both the Z and W chromosomes are expected to experience reductions in diversity compared to theoretical expectations based on N_e _alone.

Previously, we described unusual patterns of genetic diversity and recombination in a remarkable chromosomal polymorphism in the white-throated sparrow (*Zonotrichia albicollis*) that shares many characteristics of sex chromosomes [22,23]. The ZAL2 and ZAL2^m ^are heteromorphic chromosomes that differ from each other by a pair of nested inversions that span > 100 Mb and suppress recombination across the majority of the chromosome [23-25]. The inversion is of particular interest because it is linked to differences in plumage coloration, social behavior and mate choice [26]. Approximately half of the individuals in this species are homozygous for the standard arrangement (ZAL2/2) and have tan-striped (TS) crowns, while the other half of the individuals in this species are heterozygous for the inverted arrangement (ZAL2/2^m^) and have white-striped (WS) crowns [27]. In general, the TS birds display more parental behaviors than their sex-matched WS counterparts whereas the WS birds display more aggressive sexual and territorial behavior than their sex-matched TS counterparts [26,27]. The ZAL2^m ^is maintained in the population through a strong pattern of disassortative mating, where > 96% of matings are between a TS bird (ZAL2/2) and a WS bird (ZAL2/2^m^) [28]. Because the ZAL2^m ^is inherited in a Mendelian fashion, these pairings produce TS and WS offspring in equal proportions [27]. This system is analogous to the ZW, where mating pairs consist of one ZZ (male) and ZW (female) bird and produce ZZ and ZW offspring in approximately equal proportions [23,27]. Note that WS × WS breeding pairs are uncommon, and thus individuals homozygous for the inversion are rarely observed [27].

Like the Z chromosome, which recombines only in males, the ZAL2 recombines in approximately half of the population, the TS birds. In the other half, the WS birds, recombination is restricted to a small (~5-Mb) collinear segment outside the inversion that is analogous to the pseudoautosomal region of sex chromosomes [23,25]. Because no recombination occurs between the alternative chromosome arrangements within the inverted region, LD extends for > 100 Mb [22]. The unusually high LD would make the ZAL2^m ^and the ZAL2 sensitive to HRI and, in a previous study [22], we found genetic diversity within the inversion region on both arrangements to be reduced relative to the region outside the inversion. However, because sampling of the rest of the genome in our previous studies was limited, we were not able to interpret the population genetic signatures associated with the chromosomal polymorphism in the context of normal patterns of diversity and LD in this species. Furthermore, studying the patterns of variation in the white-throated sparrow genome can yield insights into the forces shaping the genetic landscape in this species, as well as those unique to avian genomes. Here we report the general population genetic patterns across the white-throated sparrow genome and evaluate those patterns in the context of chromosome size, autosomes versus sex chromosomes, and in comparison to the ZAL2/2^m ^polymorphism.

## Results

### Data set

Summary statistics by chromosome category are shown in Table 1 (with previously reported ZAL2/ZAL2^m ^data included for reference) and detailed statistics for each locus can be found in Additional file 1. In total, we sequenced ~10 kb from 27 autosomal loci (nine pairs of BAC-end sequences and nine additional loci), as well as ~6 kb from the 12 sex-linked loci, three of which were from the W chromosome (1.7 kb). The majority of the sequence was intergenic or intronic with a small fraction of protein coding positions (~2%), and thus likely to reflect neutral levels of genetic variation. For autosomal and Z-linked loci, we sampled 9 - 12 birds with a minimum of four TS and four WS birds. For W-linked loci we sampled 23 - 24 females, including at least 14 TS and nine WS birds.

**Table 1:** Sampling information and diversity values for autosomal and sex-linked loci compared to previously reported values for ZAL2 and ZAL2^m ^alternative chromosome arrangements.

*Chromosome Class*	*Orthologous chromosome in chicken*	*Number of loci sampled*	*Number of chromosomes sampled*	*Total length sampled (bp)*	*S*	*Silent π^a^*	*± SD*
Sex chromosomes	Z	9	18-22	3832 (3603)^b^	12	0.00050	± 0.00009
	W	3	46-48	1729	1	0.00005	± 0.00005
							
ZAL2^c^	3	58	16	34827 (13804)^b^	61	0.00072	± 0.00007
ZAL2^m, c^	3	58	8	34827 (13804)^b^	32	0.00039	± 0.00006
Chr2 outside of inversions^c^	3	4	24	1654 (777)^b^	17	0.00296	± 0.00041
							
Macrochromosomes	1, 2, 4	12	16-22	4773	44	0.00198	± 0.00033
Intermediate chromosomes	6, 7, 8, 9, 10	8	18-24	3101	87	0.00485	± 0.00054
Microchromosomes	17, 24, 27, 28	7	16-22	2083 (1980)^b^	136	0.01591	± 0.00115
							

*S *refers to the number of observed segregating sites.

^a^Average diversity is calculated from concatenated haplotypes from all loci in each category.

^b^Numbers in parentheses indicates the number of synonymous and noncoding positions.

^c^Data previously reported in a survey of standard ZAL2, inverted ZAL2^m ^and a region outside of the inversion polymorphism.

### Patterns of nucleotide diversity

**Figure 1:**
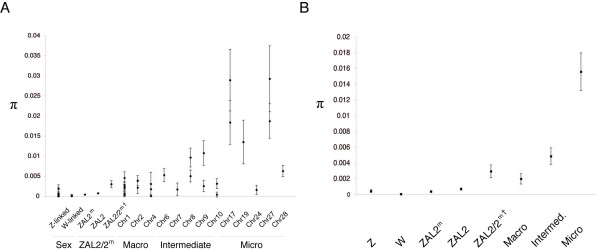
**Average diversity across the sparrow genome**. ZAL2 and ZAL2^m ^refer to average π within the inversion interval on each chromosome arrangement and ZAL2/2^m† ^indicates the region outside the inversion on white-throated sparrow chromosome 2. They are shown for reference. A. Average genetic diversity (π) per locus. B. Average genetic diversity (π) for each chromosome type is calculated from concatenated sequences from all loci within that chromosome type. Error bars represent 95% CI (± 1.96 × SD). Note that the chromosomes labeled as Chr refer to the predicted orthologous chromosome in chicken.

To understand patterns of diversity as they relate to chromosome size, we grouped our autosomal data into three categories: macrochromosomes, intermediate chromosomes, and microchromosomes. Based on the 267 identified polymorphic autosomal sites (excluding three tri-allelic SNPs), we observed a trend of increasing diversity with decreasing chromosome size (Figure 1). Overall, the amount of nucleotide polymorphism across the sparrow genome is highly variable. The nucleotide diversity across the classes of autosomes varied up to 8-fold, with the lowest diversity observed on the macrochromosomes (π ± SD = 1.98 × 10^-3 ^± 3.3 × 10^-4^) and the highest diversity on the microchromosomes (π ± SD = 1.59 × 10^-2 ^± 1.0 × 10^-3^, Figure 1B). In order to quantify the correlation between chromosome (autosome) size and genetic diversity, we used all the individual estimates of nucleotide diversity and predicted chromosome sizes based on synteny with the assembled zebra finch genome to calculate Spearman's rho, a non-parametric measure of statistical dependence, for these two variables. By this measure there was a statistically significant negative correlation between nucleotide diversity and chromosome size (rho = -0.54, p < 0.005, see also Additional files 2 and 3).

To provide independent support for the relationship we observed between nucleotide diversity and chromosome size, we calculated the pairwise divergence between available pairs of complete BAC clone sequences representing alternative haplotypes from three macrochromosomes and a microchromosome. Consistent with our results from the sequencing of multiple small loci in multiple individuals, nucleotide diversity was lower on the macrochromosomes than the sampled microchromosome. Specifically, after excluding low-quality sites and annotated protein coding exons and UTRs, the π values for the macrochromosomes were 0.00120 ± 0.0006 (145,536 sites, orthologous to chicken chromosome 1[GenBank:AC235523 and AC236253]), 0.00172 ± 0.00086 (142,469 sites, orthologous to chicken chromosome 2 [GenBank:AC237008 and AC236908]), 0.00081 ± 0.0004 (143,722 sites, orthologous to chicken chromosome 5 [GenBank:AC236607 and AC237119]), whereas the π on the sampled microchromosome was 0.01632 ± 0.00816 (106,615 sites, orthologous to chicken chromosome 17, [GenBank:AC235993 and AC235934]). Thus, in this small independent data set the highest levels of nucleotide diversity were observed on a microchromosome.

For the sex chromosomes we identified 12 Z-linked SNPs and one polymorphic site on the W chromosome. As expected, the Z and W sex chromosomes showed reduced diversity compared to the sampled autosomal loci (π ± SD = 5.0 × 10^-4 ^± 9.0 × 10^-5 ^and 5.0 × 10^-5 ^± 5.0 × 10^-5^, respectively; see Table 1 and Figure 1) and were lower than all three classes of autosomes. Our estimate of π for the W chromosome was 10-fold lower than that of the Z chromosome and between 40- and 300-fold lower than the diversity on the autosomes, depending on which chromosome type was used for comparison. Note that the high variance in the estimate of π for the W chromosome was due to the observation of just a single SNP.

Within the inversion, nucleotide diversity did not differ significantly between the ZAL2 and ZAL2^m ^(Mann-Whitney U = 1603.0, N_1 _= 58; N_2 _= 58, p = 0.575), nor did it differ from that of either sex chromosome (ZAL2/Z, MWU = 190.0, N_1 _= 58; N_2 _= 9, p = 0.123; ZAL2^m^/Z, MWU = 175.0, N_1 _= 58; N_2 _= 9, p = 0.051; ZAL2/W, MWU = 82.0, N_1 _= 58; N_2 _= 3, p = 0.836; ZAL2^m^/W, MWU = 86.0, N_1 _= 58; N_2 _= 3, p = 0.965). Because of the limited number of loci sampled on the sex chromosomes compared to the ZAL2 and ZAL2^m^, the lack of a significant difference between the inverted interval and the sex chromosomes should be interpreted with caution. Nucleotide diversity within the inversion on both the ZAL2 and ZAL2^m ^was, however, significantly lower than observed on other macrochromosomes (ZAL2: Mann-Whitney U = 123.5, N_1 _= 58; N_2 _= 12, p < 0.001; ZAL2^m^: MWU = 94.5, N_1 _= 58; N_2 _= 12, p < 0.001).

### Population structure

**Figure 2:**
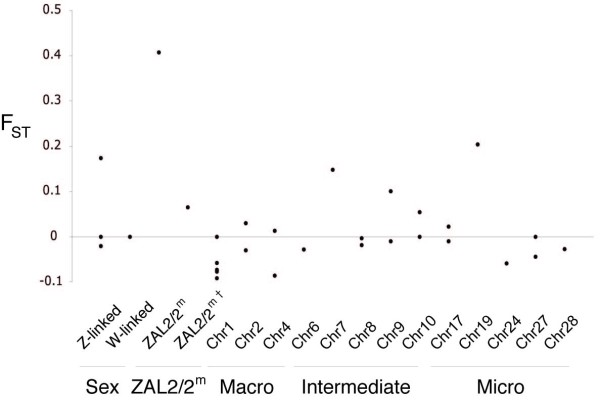
**F_ST _values between TS and WS white-throated sparrow groups across the genome**. The ZAL2/2^m ^data point represents F_ST _within the inversion interval between the arrangements and is the only value to show significant population structure (p < 0.01). The ZAL2/2^m† ^data point represents the F_ST _outside the inversion on white-throated sparrow chromosome 2. Note that the chromosomes labeled as Chr refer to the predicted orthologous chromosome in chicken. Also note the negative F_ST _values are an artifact of the method used to calculate this statistic using sequence comparisons and are not significantly different from 0.

We previously reported extreme genetic differentiation between the ZAL2 and ZAL2^m ^chromosomes as a result of suppressed recombination between the alternate chromosome types [22,23], which is also apparent in comparisons of TS and WS individuals. In order to compare this genetic differentiation to that present elsewhere in the genome, we calculated F_ST _between the groups of TS and WS birds at all the sampled loci (Figure 2 and Additional file 1). Most F_ST _values for the autosomal and sex-linked loci clustered around zero, and we found no significant signal of population structure across the genome except within the ZAL2/2^m ^inversion interval (Figure 2). Additionally, none of the haplotype networks constructed from individual loci unlinked to the ZAL2/2^m ^polymorphism revealed any clear indication of population structure (data not shown), indicating the genetic differentiation observed between TS and WS birds within the ZAL2/2^m ^inversion is exceptional with respect to the rest of the genome.

### Allele frequency spectra

**Figure 3:**
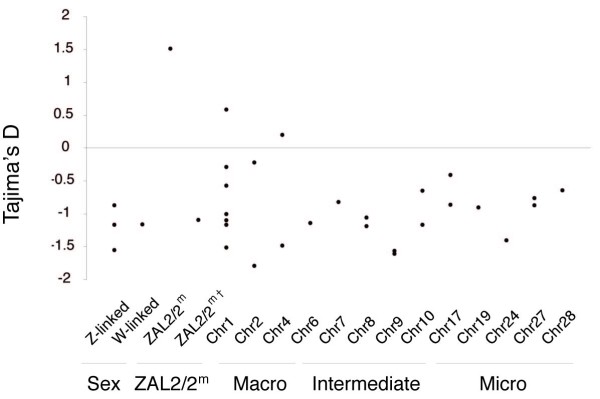
**Tajima's D values across the sparrow genome**. The ZAL2/2^m ^data point represents data from within the inversion on both chromosome arrangements, while the ZAL2/2^m† ^represents Tajima's D for outside the inversion on white-throated sparrow chromosome 2. Note that the chromosomes labeled as Chr refer to the predicted orthologous chromosome in chicken.

Tests based on allele frequency distributions can yield insights into demographic, population genetic and evolutionary processes acting upon a particular genomic region. Previously, we reported a skew towards intermediate frequency alleles within the ZAL2/ZAL2^m ^system and a positive Tajima's D [22,23]. To establish whether this pattern was unique to the ZAL2/ZAL2^m ^system within the sparrow genome, we calculated Tajima's D for all sampled loci (Figure 3, Additional file 1). As with the F_ST _values, the Tajima's D associated with the ZAL2/ZAL2^m ^chromosomal polymorphism was an outlier compared to the other regions of the white-throated sparrow genome. No Tajima's D values were found to be statistically different from neutral expectations, owing greatly to the conservative nature of the statistic. However, a one-tailed t-test demonstrates that the Tajima's D value for the ZAL2/2^m ^system is statistically different from values reported elsewhere in the genome (t = 27.72, p < 0.0001).

### Linkage disequilibrium and recombination

**Figure 4:**
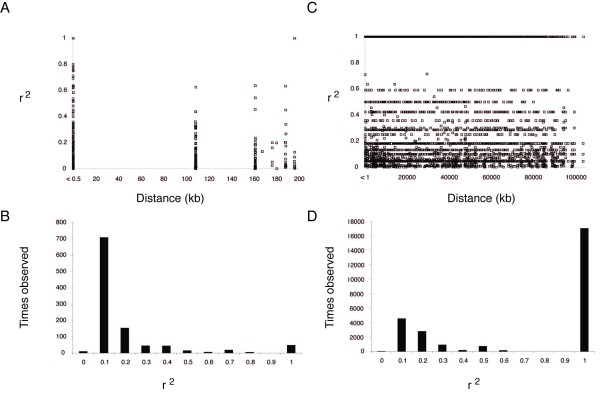
**Linkage disequilibrium patterns within the sparrow genome**. A. Patterns of LD between alleles among autosomal loci (excluding ZAL2/2^m ^data) show that LD is generally very low and decreases slightly with distance (See Additional file 4). B. A histogram indicates that the majority of pairwise comparisons among autosomal loci have 0 < r^2 ^≤ 0.1. C. Patterns of LD between alleles among ZAL2/2^m ^loci show high LD spanning > 100 Mb. D. A histogram indicates that the vast majority of pairwise comparisons within the ZAL2/2^m ^inversion show high r^2^, 0.9 < r^2 ^≤ 1.

Recombination is expected to reduce LD and, in general, LD decreases with increasing distance between sites. To compare LD associated with the ZAL2/2^m ^chromosomal polymorphism to that observed in the rest of the genome, we pooled r^2 ^values from informative pairwise comparisons within autosomal loci, plotted their distribution as a function of distance (Figure 4A) and calculated their averages (Additional file 4). The r^2 ^values from our autosomal data were generally low, as measured by ZnS = 0.14, a summary statistic which is based on the average correlation between pairs of sites [29]. Among the autosomal sites, LD decayed rapidly such that only limited LD was observed even within 500 bp (Figure 4A and 4B). Consistent with the overall low levels of LD, the four-gamete test revealed evidence for recombination across all our autosomal BAC-paired end loci (data not shown). Note that due to the limited number of informative pairwise comparisons on the Z (n = 3), no similar analyses could be performed on that chromosome. In contrast, we previously observed perfect LD between the majority of pairwise comparisons within the ZAL2/ZAL2^m ^inversion, independent of distance between sites and extending to > 100 Mb (Figure 4C and 4D). Thus, the strong LD associated with the chromosomal polymorphism was exceptional compared to other white-throated sparrow chromosomes.

## Discussion

Nucleotide variation, linkage disequilibrium and genetic structure are all fundamental parameters that describe the population genetics of a species. Initial studies in the white-throated sparrow focused on characterizing the unusual ZAL2 and ZAL2^m ^chromosome polymorphism [22,23]. In this study, we sequenced loci from other autosomes, as well as sex chromosomes to understand population genetic patterns elsewhere in the genome and to compare and contrast these patterns with those previously reported for the ZAL2 and ZAL2^m ^chromosomes.

### Contrasting patterns of nucleotide diversity within the sparrow genome

On average across all sparrow autosomes, one segregating site is observed every ~180 bp (π = 5.62 × 10^-3 ^± 4.7 × 10^-4^). This estimate is similar to the diversities calculated in other natural populations of passerine birds, such as *Ficedula *flycatchers, π = 2.7 - 3.6 × 10^-3^, [20,30], *Carpodacus *finches, π = 5.7 - 8.5 × 10^-3 ^[31], the great reed warbler, π = 1.2 × 10^-3 ^[32] and the blue tit, π = 1.8 × 10^-3 ^[32]. Nucleotide diversity across the sparrow autosomes varies substantially, spanning a range of nearly two orders of magnitude between individual loci (π = 3.0 × 10^-4 ^- 2.9 × 10^-2^). The microchromosome group on average was associated with the highest level of nucleotide diversity and was 8-fold higher than the macrochromosomes. However, as pointed out in the Results section, the level of nucleotide diversity was not perfectly correlated with chromosome size. For example, the diversity of the loci orthologous to microchromosomes 24 and 28 were comparable to that observed on the macrochromosomes. This result could be attributed to a number of factors, such as local variation in recombination rates, or may reflect inaccurate assignment of these white-throated loci to chromosome class sizes caused by differences in genome organization between this species and the zebra finch or chicken.

The negative correlation between nucleotide diversity and chromosome size class observed in our study was previously predicted for avian genomes based on two well-established observations. The first is that the rate of recombination on avian microchromosomes can be 5-10 times greater than on macrochromosomes [9,10]. The second observation is that regions of high recombination tend to harbor greater genetic variation [7]. Nevertheless, previous studies that tested for this correlation in nucleotide diversity in the chicken genome reported conflicting results [13,14,33]. Future studies will therefore be necessary to establish whether the negative correlation between nucleotide diversity and chromosome size we observed for the white-throated sparrow autosomes is a general characteristic of avian genomes.

As expected, the white-throated sparrow sex chromosomes showed the lowest levels of genetic diversity. The neutral theory predicts a ratio of 1:3:4 for the genetic diversities of the W, Z and autosomes based on their relative N_e_s. Z to autosome diversity (π_Z _: π_A_) is therefore expected to equal 0.75 [21]. However, because sex chromosomes are transmitted differently from autosomes, the standard models of how mutation, selection, drift, demography and mating system influence population genetic patterns may not apply [15]. The combined effects of increased HRI and lowered N_e _due to variation in male mating success can reduce Z chromosome diversity relative to autosomal diversity [20,21]. Indeed, reports of avian π_Z _: π_A _are lower than the expected 0.75: in *Ficedula *flycatchers π_Z_: π_A _≈ 0.4 [20] and in chicken π_Z_: π_A _≈ 0.25 [21]. However, because those studies did not indicate the chromosome size class of the autosomal loci used for comparison, the degree to which the π_Z_: π_A _is skewed is uncertain. Our finding that chromosome size and genetic diversity are negatively correlated suggests that it is appropriate to consider Z chromosome diversity in reference to similarly sized chromosomes (macrochromosomes). In the white-throated sparrow the π_Z_: π_A _≈ 0.08 when comparing Z chromosome diversity to average diversity across all autosomes. However, when comparing Z chromosome diversity to average macrochromosome diversity, π_Z_: π_A _≈ 0.25, which highlights the importance of considering chromosome type when calculating this ratio.

Under the neutral model, the W chromosome-to-autosome diversity is predicted to be 1:4 or 0.25. However, studies of the avian W chromosomes report far lower diversity values. For example, in a survey of ~3.4 kb from > 150 W chromosomes in seven avian species, Montell et al. [17] did not observe any polymorphisms. Berlin and Ellegren [16] identified a single segregating site in a survey of ~8 kb in 47 chickens from divergent breeds and estimated π_W _= 7 × 10^-5^, ~1/100 of their π_A _estimate [21]. Similarly, we observed low levels of variation on the W chromosome, although it should be pointed out that our estimate of π for the W was based on a single SNP.

Within the white-throated sparrow genome, dramatic HRI is probably not limited to the sex chromosomes. We previously reported patterns of reduced recombination and extensive LD within the ZAL2/2^m ^system, which bears striking similarities to sex chromosomes [22,23]. Avian macrochromosomes are disproportionately associated with recombination deserts, except close to the chromosome ends [10]. Thus, the low rate of recombination for macrochromosomes is likely to result in intrinsically low diversity values, which are further reduced by HRI on the ZAL2 and ZAL2^m^.

Although the ZAL2/2^m ^system bears many similarities to the XY (ZW) sex chromosomes, including differences in patterns of recombination, disassortative mating, and reduced genetic diversity, the ZAL2/2^m ^are not sex-linked and, as such, sexual selection [34], sex-biased demographic history [35,36], nor mitochondrial linkage [19] can easily explain patterns of evolution within the ZAL2/2^m ^system. For these reasons, the ZAL2/2^m ^polymorphism remains an informative point of comparison for the study of population genetic patterns of sex chromosomes and can help distinguish between sex-specific and fundamental molecular evolutionary processes that shape their evolution.

### LD within the sparrow genome

The extent of LD is dependent on two key parameters: N_e _and recombination rate [9,37]. While the extent of LD within avian genomes from natural bird populations is largely unknown, the few studies currently available indicate that LD is generally low on avian autosomes as a consequence of high rates of recombination [33,38-40]. Megens *et al*. [33] reported a correlation between LD and chromosome size, such that macrochromosomes exhibit consistently higher LD when compared to microchromosomes. Our autosomal data are consistent with these reports in that on average we found only weak LD even between sites within a few hundred bases of each other, and that the macrochromosomes were associated with the strongest LD (see Additional file 4).

The one striking exception to the general pattern of low LD in the sparrow genome we did observe was within the ZAL2/ZAL2^m ^inversion. Our previous study of the ZAL2 and ZAL2^m ^chromosomes revealed extensive LD spanning > 100 Mb, thus representing one of the largest segregating haplotypes ever reported [22]. Given our findings here, as well as the general patterns of low LD reported in other bird autosomes [33,38,39], we conclude that the LD observed between the ZAL2 and ZAL2^m ^is unusual within the sparrow genome as well as amongst avian genomes in general.

### Population structure

Our previous studies of the ZAL2/2^m ^system indicated extensive population structure within the inversion polymorphism [22,23]. The majority of the segregating sites (~70%) were fixed differences and F_ST _= 0.94 (p < 0.01) within the inversion between the alternate arrangements [22]. The high proportion of fixed differences between the chromosome arrangements resulted in many intermediate frequency variants, and thus a large and positive Tajima's D value. In this study of autosomal and sex-linked loci, we found no significant population structure between WS and TS birds outside of the ZAL2/2^m ^inversion. Additionally, the negative Tajima's D values observed for most of the other sampled loci indicate that the effects of balancing selection through disassortative mating are limited to the ZAL2/2^m ^system.

## Conclusions

Our population-based sequencing survey of autosomes and sex chromosomes in the white-throated sparrow provides empirical evidence for the predicted negative correlation between chromosome size and nucleotide diversity in avian genomes. In addition, we found that the patterns of nucleotide diversity, population structure, and LD previously associated with the ZAL2/2^m ^chromosomal polymorphism in this species are atypical compared to other macrochromosomes.

## Methods

### White-throated sparrow samples

White-throated sparrows and a female dark-eyed junco (*Junco hyemalis*) were collected in mist nets on the campus of Emory University in Atlanta, GA during November and December of 2005-2008. Visual examination of the plumage and a PCR-based assay were used to infer the genotype with respect to the ZAL2/ZAL2^m ^polymorphism for each bird included in the study [41]. Note that the frequency of WS and TS individuals in our locally sampled population is comparable to the overall frequency reported for the species, *i.e*. 50:50 (DL Maney, unpublished data). The Emory University Institutional Animal Care and Use Committee approved all procedures involving animals.

### DNA sequencing

PCR primers for autosomal and Z chromosomes were designed in Primer3 [42] using white-throated sparrow BAC-end sequences that mapped to unique locations in the zebra finch genome (taeGut1)[43] by MEGABLAST searches (-t 16, -N 2, -W 11, -e 1e-30) [44]. For the W-linked loci, we designed primers from a completely sequenced white-throated sparrow BAC from the W chromosome [GenBank: AC236562] containing the *CHD1W *locus. To avoid potential amplification of gametologous Z chromosome loci, the W chromosome primers were designed to include at least three mismatches compared to the corresponding Z chromosome sequence. A complete list of PCR primers, their orthologous positions in zebra finch, and orthologous chromosome assignments in chicken are listed in Additional file 5. Each 25 μL PCR contained final concentrations of 1X PCR buffer, 1.5 mM MgCl_2_, 20 pM of each primer, 0.2 mM of each dNTP, 1.5 U of Taq or Platinum Taq DNA polymerase (Invitrogen) and ~25 ng of genomic DNA. PCR cycling parameters were as follows: 94° for 5 min, 35 cycles of 94° for 30 sec, 55° for 30 sec and 72° for 1 min, followed by 72° for 7 min. Amplicons were subsequently purified and directly sequenced (Sanger sequencing) [GenBank: GU449819 - GU450219, GU450273 - GU450309 and HM126554 - HM126573].

### SNP discovery and sequence annotation

Nucleotide polymorphisms were automatically called using SNPdetector [45] and manually confirmed prior to further analyses. Annotation of the gene features for each locus was based on the annotation of orthologous zebra finch genomic segments (taeGut1) [43]. Insertion-deletion polymorphisms and sites with more than two segregating alleles were excluded from our analyses, as these events were relatively infrequent and could not be readily incorporated into the subsequent analyses. Like most other avian genomes, the white-throated sparrow karyotype consists of macro-, intermediate and microchromosomes [24]. Given that synteny is highly conserved among even distantly related birds, such as the zebra finch and chicken [43], we predicted linkage and corresponding chromosome size in the white-throated sparrow based on orthology to the zebra finch and chicken genomes. To examine population genetic patterns between differently sized autosomes, we grouped our sequence data by chromosome type according to the classification convention of the International Chicken Genome Sequencing Consortium that divides chicken autosomes into three size classes: macrochromosomes (GGA 1-5), intermediate chromosomes (GGA 6-10) and microchromosomes (GGA 11-38) [9].

### Population genetic analysis

Where we had sequence from paired BAC ends, genotypes were concatenated and phased as a single locus using Phase v2.1.1 [46]. Haplotypes were then split into individual loci for population genetic analysis, except for calculation of LD and construction of haplotype networks. Analysis of polymorphism and tests of neutrality based on allele frequency spectrum, (Tajima's D and Fu and Li's statistics) were performed in DnaSP v5.1 [47].

Using Splitstree v4.10 [48], we generated haplotype networks using the neighbor-joining algorithm and *J. hyemalis *sequence as an outgroup. Haplotype networks were constructed for individual loci and, when possible, for the concatenated paired BAC-ends. To quantify population structure, F_ST _values were calculated in Arlequin v3.11 [49] and statistical significance was assessed with exact tests for genetic differentiation. In order to make a direct comparison between F_ST _within the ZAL2/ZAL2^m ^system and the autosomal and sex-linked loci reported here, we calculated F_ST _between the TS and WS groups. LD and r^2 ^values for all pairwise comparisons between informative sites, including paired BAC-end sequences when possible, were calculated with Haploview v4.2 [50]. In addition to inferring recombination from levels of LD, we identified four-gamete pairs within all loci using DnaSP v5.1 [47] and estimated the average haplotype block size using the four-gamete rule in Haploview v4.2 [50].

In order to compare levels of diversity among the ZAL2/2 m, sex chromosomes, and macrochromosomes we performed an overall omnibus test followed by planned pairwise comparisons. Because the data set contained many zeros, the data could not be normalized by transformation. We therefore used a nonparametric approach (Kruskal-Wallis ANOVA followed by Mann-Whitney U tests).

## Authors' contributions

LYH, DLM and JWT conceived the project. LYH performed the genotyping and population genetic analyses. DLM provided the DNA samples. LYH and JWT drafted the manuscript and all authors approved the final version of the manuscript.

## Supplementary Material

Additional file 1 **Supplementary Table 1**. Population genetics statistics for each locus in the study. Click here for file

Additional file 2 **Supplementary Figure 1**. Sparrow genetic diversity by chromosome size. Click here for file

Additional file 3 **Figure legend for Supplementary Figure 1**. Click here for file

Additional file 4 **Supplementary Table 2**. Summary statistics for linkage disequilibrium by chromosome type and distance. Click here for file

Additional file 5 **Supplementary Table 3**. PCR primer information for all loci in this study. Click here for file
