# Supplementary figures and images for: Contrasting population genetic patterns within the white-throated sparrow genome (Zonotrichia albicollis)
Source: BMC Genet. 2010 Oct 28;11:96. doi: 10.1186/1471-2156-11-96 (PMC3223602; doi:10.1186/1471-2156-11-96)

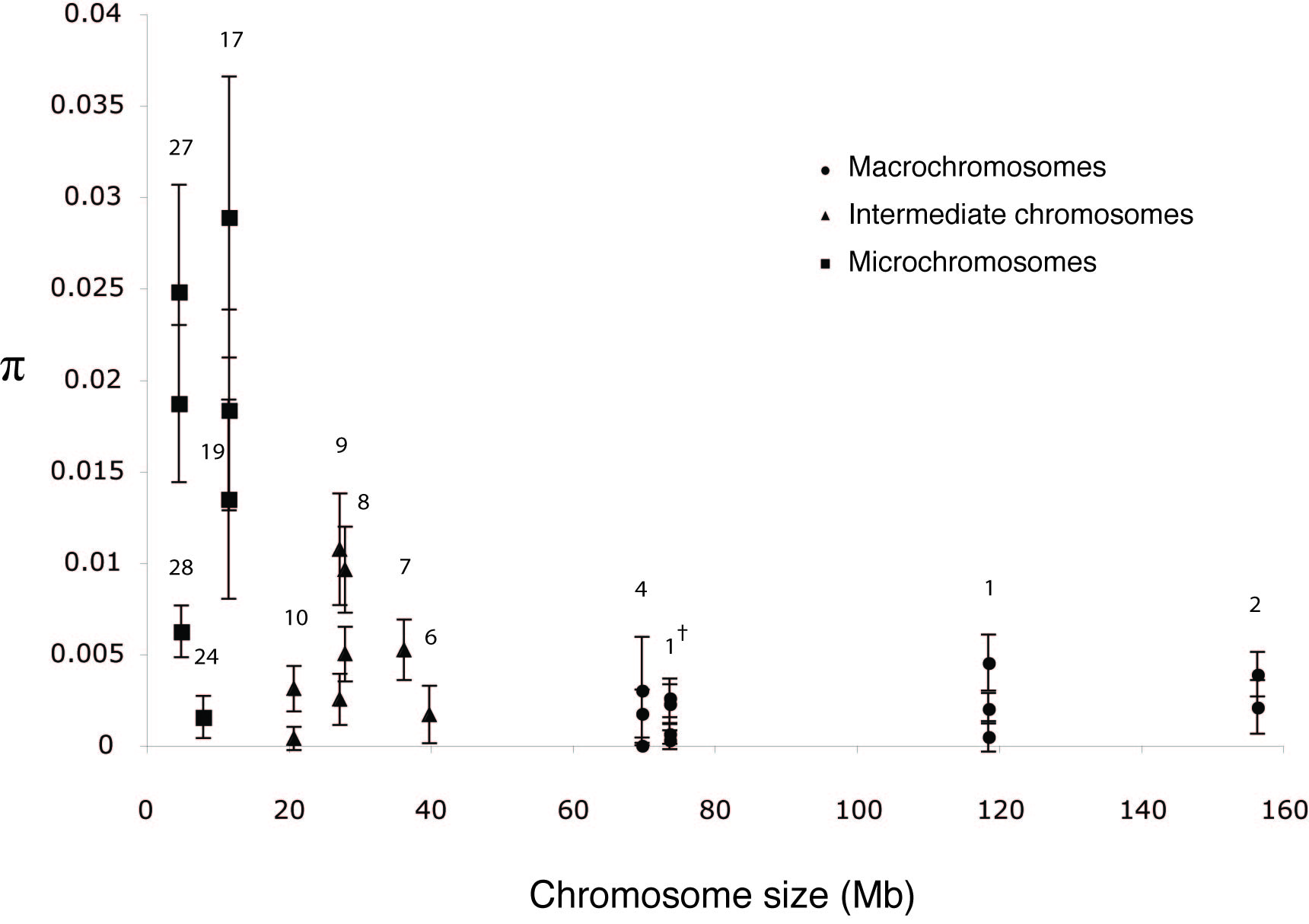

Supplement: Additional file 2 — Supplementary Figure 1. Sparrow genetic diversity by chromosome size. [file 1471-2156-11-96-S2.JPEG]
