# Supplementary material for: Contrasting population genetic patterns within the white-throated sparrow genome (Zonotrichia albicollis)
Source: BMC Genet. 2010 Oct 28;11:96. doi: 10.1186/1471-2156-11-96 (PMC3223602; doi:10.1186/1471-2156-11-96)
Supplement: Additional file 3 — Figure legend for Supplementary Figure 1. [file 1471-2156-11-96-S3.DOC]

**Supplemental Figure 1. Chromosome size and genetic diversity show a negative correlation across the white-throated sparrow genome.** Numbers above data points indicate orthologous chicken/zebra finch chromosome as assigned in the genome sequence assemblies (Warren et al, 2010, Hillier et al, 2004). The chromosome sizes are based on the zebra finch genome assembly, which as a Passerine, is more closely related to the white-throated sparrow than the chicken. Chicken chromosome 1 corresponds to zebra finch chr1A (indicated by †) and chr1 in zebra finch.
